# Barefoot doctors: A solution to the dilemma of COVID-19 prevention and control in rural areas of economically underdeveloped countries

**DOI:** 10.3389/fpubh.2022.1020312

**Published:** 2022-11-04

**Authors:** Shi-Wei Hu

**Affiliations:** The First Clinical Medical College of Xuzhou Medical University, Xuzhou, China

**Keywords:** COVID-19, infectious disease prevention and control, barefoot doctors, rural doctor, barefoot doctor training

## Introduction

Many less developed countries lack doctors, especially in rural areas. Nigeria, for example, is a poor country, as evidenced by its extremely low per capita income. In rural areas of the country, about 22,000 people are equipped with a doctor, while in towns and cities, about 12,000 Nigerians are equipped with a doctor (1). Nigeria's doctor population ratio is well-below the World Health Organization's recommendation of 1:1,000. Thus it can be said that Nigeria's health care base is very weak, and the situation is particularly bad in rural areas. This is just a microcosm of rural health care in less developed countries. Many low-income countries, including Nigeria, are failing to meet the basic health care needs of their people, especially in rural areas (2). Since the outbreak began around the world, the global economic downturn caused by the pandemic has further strained national finances in less developed countries (3). In order to prevent the domestic economy from further declining due to the epidemic, less developed countries had to cut the health financial expenditure in rural areas to protect the public health in cities. Futhermore, people's health awareness in rural areas, especially the awareness of infectious disease prevention and control is very weak (4). This has made rural areas in some countries the hardest hit by the pandemic. Addressing COVID-19 in rural areas is an integral part of the global COVID-19 response. The barefoot doctor model, which successfully solved the problem of epidemic in the early years of New China in the last century, may be able to solve the current dilemma of epidemic prevention and control in rural areas.

## Past and present of barefoot doctor

### The development of the barefoot doctor

**Figure 1:**
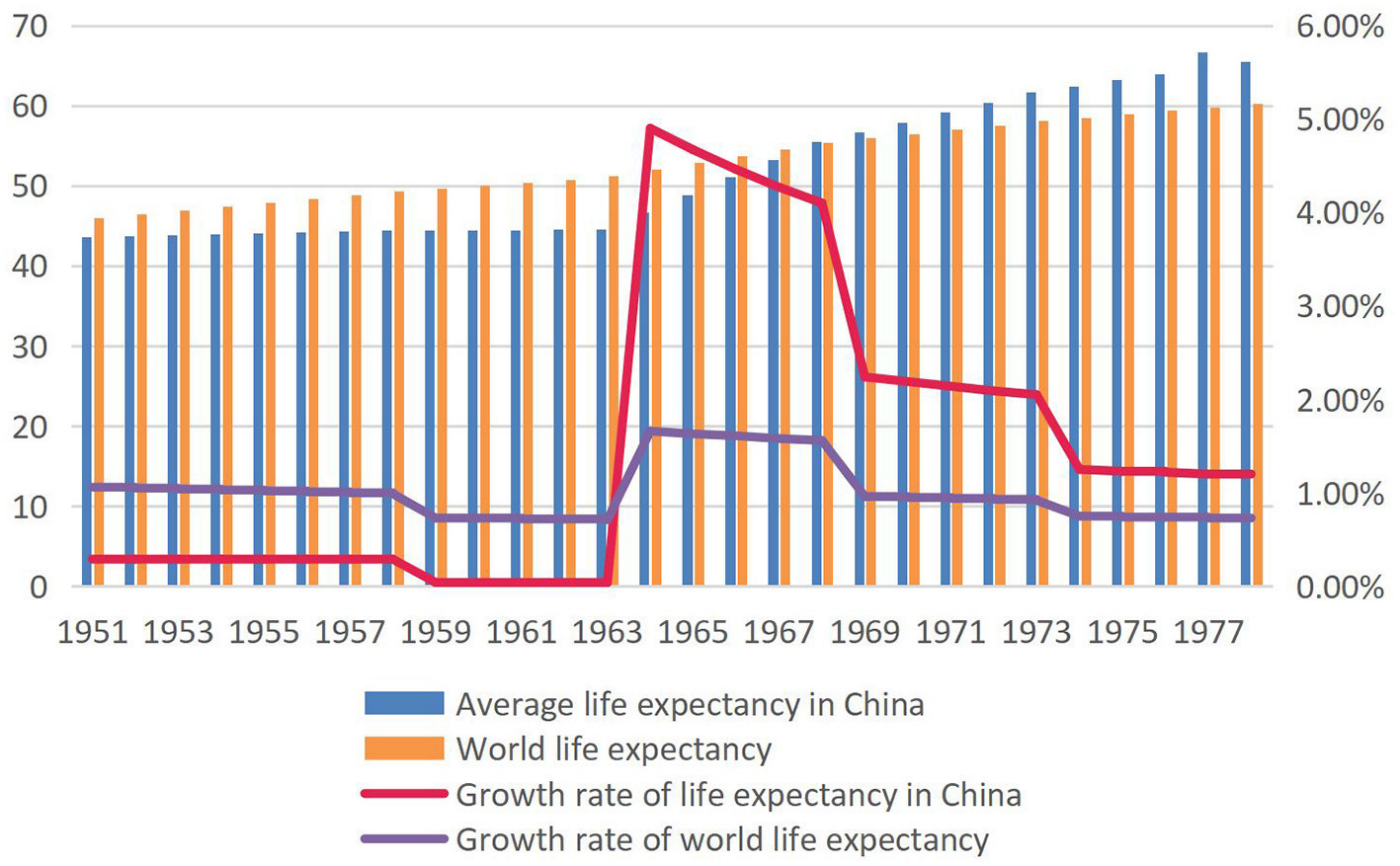
Average life expectancy and growth rate of the world and China, 1951–1978 (16, 17). The average life expectancy in China was lower than the world average life expectancy in 1951 and the gap gradually widened between 1951 and 1963. Since 1964, the growth rate of China's average life expectancy has risen rapidly, far higher than that of the world. In 1967, China's average life expectancy was equal to the world's average life expectancy.

Barefoot doctors are medical personnel with certain medical knowledge and ability who emerged in China in the 1960's and 1970's without fixed staffing authorization and were generally approved and appointed by rural or grass-roots governments. As they are both farmers and doctors, working in busy times and practicing medicine in slack times, the villagers colloquially call those barefoot doctors who work in the fields barefoot (5). The emergence of barefoot doctors in China has a unique historical background. After the founding of People's Republic of China, the Communist Party of China and the Chinese government attached great importance to the improvement of rural health conditions. Although medical and health care in rural areas has been improved to a certain extent, the chronic shortage of medical personnel in rural areas has not been fundamentally resolved. In 1965, “there were over 1.4 million medical staff in China. Eighty per cent of senior medical staff were in cities, 70 per cent in big cities, 20 per cent in county towns and only 10 per cent in rural areas (6). The budget, including the current rural areas, was 25 percent, and 75 percent went to cities (7).” In the early 1960's, many high-ranking party officials deplored the serious problems in rural medical service after returning from their trips to rural areas during the socialist education campaign (8). The Ministry of Health, which Mao derided as “the health ministry for lords,” acted immediately by training many local peasants as part-time health workers who were referred to as “barefoot doctors” (9). The emergence of barefoot doctors was China's choice between the best plan and the feasible plan, that is to say, in a short time and with less effort, to train some staff with a certain amount of medical skills, but greatly needed in rural areas. From the economic perspective, since the founding of the People's Republic of China in the 1960's, the economy was poor. In 1963, for example, the nominal monetary value of China's total health expenditure was 3.117 billion yuan, or 4.57 yuan per capita (10). During the same period in 1960, the total health expenditure in the United States had reached 27.1 billion US dollars, and the per capita health expenditure was 149.72 US dollars. They were about 66.72 billion yuan and 368.59 yuan, respectively according to the exchange rate at that time (11). The total health expenditure in China was only 4.7 and 1.2% of that in the United States, respectively. What's more, the rural areas only accounted for 25% of the expenditure during that time. Compared with the cities, the medical and health conditions in the rural areas of China were more backward in the 1950's and 1960's. The farmers did not have enough money to see a doctor, and the rural areas did not have enough financial support to train professional doctors. In addition, the years of war before the founding of the People's Republic of China led to rampant epidemics, and the average life expectancy was only 40 years old (12). In fact, the way doctors are trained depends on the corresponding socioeconomic and technological level. Before the 18th century, the “general doctors” in Europe and The United States were only apprentices trained as healers (13). The eventual spread of barefoot doctors across the country overcame many difficulties, such as superstition about witch doctors, lack of funding, villagers' doubts about the competence of health professionals, and conflicts of interest at different levels (doctors in regular hospitals look down on barefoot doctors) (14). The belief in witch doctors was the most serious. Large numbers of people refused to be treated by the government's mobile medical teams and turned to shamans for help. Even some cadres who accepted atheism performed supernatural healing when their family members fell ill (15). The government has tried many ways to rid the countryside of the belief in witch doctors, but with little success. The result was that the barefoot doctor and the witch doctor worked together, leaving each other alone. Barefoot doctors alleviated the serious public health crisis in China at the minimum cost, and strictly controlled malaria, smallpox, schistosomiasis and other infectious diseases. The clearest evidence of this is the rapid rise in life expectancy in China, well-above the world average, since the 1960's, when a large number of barefoot doctors were trained (Figure 1). In 1985, barefoot doctors were removed from the medical profession in China, and those who passed the examination were renamed rural doctors. Rural doctors still play an important role in the primary health service system in rural China today (9).

### The role of barefoot doctors in COVID-19 prevention and control

As the main force of rural medical and health care, barefoot doctors mainly assume the following responsibilities: 1 health and epidemic prevention responsibility, 2 diagnosis and treatment responsibility, 3 health education and psychological consultation (18). The barefoot doctor's first task is sanitation and epidemic prevention because treatment costs far more medical resources than prevention. Nevertheless, poor areas are often faced with the problem of shortage of medical resources, such as lack of funds, lack of drugs and too many patients (19). Barefoot doctors are the key to ensuring the health of as many people as possible with very limited medical resources. Barefoot doctors improve sanitation in rural areas by spraying preventive medicine regularly and building public toilets that meet hygienic standards to prevent the spread of disease. During the COVID-19 pandemic, barefoot doctors are also responsible for the diagnosis and treatment of common and frequently occurring diseases in rural areas as well as delivering babies.

### Barefoot doctors offer new ideas for addressing public health challenges in less developed countries

Two years into the COVID-19 pandemic, the spread of monkeypox virus in many countries around the world in 2022 has exacerbated the public health crisis in underdeveloped countries (20). Healthcare systems in many countries are on the verge of collapse, and even in some developed countries, medical resources are stretched. When poor countries face infectious disease pandemics, they cannot rely on medical assistance from developed countries alone. Adopting an economically feasible and efficient medical and health system may be the best choice to deal with the epidemic. After all, developed countries also have to deal with the epidemic in their own country first when the epidemic is spreading; meanwhile the underdeveloped countries may have already missed the best time to prevent the spread of the epidemic when they receive assistance from developed countries. Some people are opposed to the idea that amateur doctors without professional medical training can take on the burden of epidemic prevention and control. Indeed, barefoot doctors may not stop outbreaks, but they can reduce the harm and slow down the spread of diseases. Barefoot doctors understand the lifestyles and habits of people in their own community, and they are more likely to gain people's trust than migrant medical staff. They also know how to treat different groups of people, and timely pass on the correct information about COVID-19 to other members of the community. Without professional medical knowledge and practice, they can only provide basic and simple medical services, such as conveying hygiene concepts such as “washing hands before eating” to the public, administering vaccinations and distributing anti-epidemic drugs. When these seemingly trivial measures are widely adopted, they can create qualitative changes and form a strong public health safety barrier. Rural areas in less developed countries can also consider combining low-cost traditional medicine with higher-cost western medicine to save medical resources on the premise of ensuring the health of patients. Considering the low educational level of rural people in underdeveloped countries (21), barefoot doctors are also responsible for promoting health knowledge, encouraging people to abandon bad customs, and providing psychological comfort to patients. In fact, as early as the 1970's, barefoot doctors in China have already attracted the attention of the United Nations World Health Organization. In its 1980–1981 annual report, the United Nations Fund for Women and Children said that China's “barefoot doctor” model provided primary care for underdeveloped rural areas and provided a model of improving health care in underdeveloped countries (22).

## Discussion

As rural population mobility is far less than urban population mobility (23), people tend to only care about the prevention and control of COVID-19 in big cities. Nonetheless, the epidemic prevention and control in rural areas is also of paramount importance. Due to the backward infrastructure and lack of primary medical workers in rural areas of economically underdeveloped countries, it is difficult to timely collect COVID-19 data in rural areas and prevent the spread of the epidemic once it occurs. At present, rural areas in less developed countries need a large number of medical workers with certain knowledge and skills of infectious disease prevention and control. It is a good choice to follow the “barefoot doctor” model of China in the last century. Besides, the training of barefoot doctors should be timely and targeted according to the disease spectrum and epidemic diseases in the region. Localization is especially important in the training of barefoot doctors. Barefoot doctors in a village tend to form a more harmonious doctor-patient relationship because of neighborhood relations (24). It is very beneficial for barefoot doctors to carry out a series of educational campaigns and prevention and control of infectious diseases. Nowadays, it is difficult for a barefoot doctor to take root in rural areas only with a modest salary. It is also necessary to skillfully use ideological and political education and improve the social status of barefoot doctors to stimulate the spirit of barefoot doctors, and shape the spirit of selfless dedication for the people and the motherland (25).

## Author contributions

S-WH conceptualized and wrote the manuscript.

## Conflict of interest

The author declares that the research was conducted in the absence of any commercial or financial relationships that could be construed as a potential conflict of interest.

## Publisher's note

All claims expressed in this article are solely those of the authors and do not necessarily represent those of their affiliated organizations, or those of the publisher, the editors and the reviewers. Any product that may be evaluated in this article, or claim that may be made by its manufacturer, is not guaranteed or endorsed by the publisher.

## References

[1] AllAfrica.com. Nigeria Has Only 42,000 Doctors to 200 Million People, NMA President Cries Out. Available online at: https://allafrica.com/stories/201912190053.html (accessed April 23, 2020).

[2] Omonona BT Obisesan AA Aromolaran OA. Health-care access and utilization among rural households in Nigeria. J Dev Agric Econ. (2015) 7:195–203. 10.5897/JDAE2014.0620

[3] Rasul G Nepal AK Hussain A Maharjan A Joshi S Lama A. Socio-economic implications of COVID-19 pandemic in South Asia: emerging risks and growing challenges. Front Sociol. (2021) 6:629693. 10.3389/fsoc.2021.629693 33869579 PMC8022444

[4] Ma L Liu H Tao Z Jiang N Wang S Jiang X. Beliefs/attitudes, and practices of rural residents in the prevention and control of COVID-19: an online questionnaire survey. Am J Trop Med Hyg. (2020) 103:2357–67. 10.4269/ajtmh.20-0314 33124537 PMC7695081

[5] Wei CN. Barefoot doctors: the legacy of chairman Mao's healthcare. Mr. Science and Chairman Mao's Cultural Revolution (Plymouth: Lexington Books Press) (2013). 251–80.

[6] Qian X. Conveying the Spirit of the Chairman's Instruction in the Director's Group of the National Rural Medical Education Conference Hall“ (a Collection of Conference Materials Issued by the Secretariat of the National Rural Medical Education Conference in 1965). Beijing: People's Medical Publishing House (1965).

[7] Yi-Qun W. Social and cultural factors of the emergence and existence of Barefoot doctors. J Yunnan Minzu Univ. (2005) 2:60–3. 10.13727/j.cnki.53-1191/c.2005.02.013

[8] Yang S. Yang Shang-kun's Diaries. Beijing: Central Literature Publishing House (2001).

[9] Xu S Hu D. Barefoot doctors and the “health care revolution” in rural China: a study centered on Shandong Province. Endeavour. (2017) 41:136–45. 10.1016/j.endeavour.2017.06.004 28693889

[10] Le-Xun D Yu-Xin Z Guo-Xiang L. Review and prospect of government health investment and total health cost accounting in the 60 years since the founding of the People's Republic of China. Health Policy Res China. (2009) 2:15–20.

[11] Chinese Health and Family Planning Yearbook. China Health and Family Planning Yearbook. Beijing: Chinese Health and Family Planning Yearbook (2016).

[12] Statista. Life Expectancy (from birth) in China From 1850 to 2020. (2022). Available online at https://www.statista.com/statistics/1041350/life-expectancy-china-all-time/ (accessed October 16, 2022).

[13] Chun-Rong W. The origins of general practice. Chinese J Gen Pract. (2002) 2:45–7. 10.3760/cma.j.issn.1671-7368.2002.02.016

[14] Zhou X. Reconsidering the barefoot doctor Programme. Fudan J Humanit Soc Sci. (2016) 9:41–63. 10.1007/s40647-015-0107-6

[15] Zhou X. Great Famine in China, 1958–1962: A Documentary History. New Haven, London: Yale University Press (2012).

[16] MacrotrendsLLC. WorldLife Expectancy 1950-2022. (2022). Available online at: https://www.macrotrends.net/countries/WLD/world/life-expectancy (accessed October 14, 2022).

[17] MacrotrendsLLC. China Life Expectancy 1950-2022. (2022). Available online at: https://www.macrotrends.net/countries/CHN/china/life-expectancy (accessed October 14, 2022).

[18] Sha-Sha Z Qi-Lian L Xiao-Min X. The prototype of general practitioner in China – barefoot doctor. Prim Health Care China. (2022) 36:7–10. 10.3969/j.issn.1001-568X.2022.05.0003

[19] Arize I Ogbuabor D Mbachu C Etiaba E Uzochukwu B Onwujekwe O. Stakeholders' perspectives on the unmet needs and health priorities of the urban poor in South-East Nigeria. Int Q Community Health Educ. (2021) 0272684X211033441. 10.1177/0272684X211033441 34264139

[20] Shanmugaraj B Khorattanakulchai N Phoolcharoen W. Emergence of monkeypox: another concern amidst COVID-19 crisis. Asian Pac J Trop Med. (2022) 15:193. 10.4103/1995-7645.346081

[21] Castañeda A Doan D Newhouse D Nguyen MC Uematsu H Azevedo JP. A new profile of the global poor. World Dev. (2018) 101:250–67. 10.1016/j.worlddev.2017.08.002

[22] United Nations Children's Fund. The state of the world's children 1981–1982. State of the World's Children. (1982) 5:8. 10.18356/a4d964a5-en

[23] Wang H Zhang M Li R Zhong O Johnstone H Zhou H. Tracking the effects of COVID-19 in rural China over time. Int J Equity Health. (2021) 20:35. 10.1186/s12939-020-01369-z 33446205 PMC7807215

[24] Kui-Li Z. Barefoot doctors and community doctor-patient relationship: based on social capital theory. Study Soc. (2014) 6:119–27.

[25] Hu Y. “China's road”: the cooperative medical services as a “paradigm”. Rural Health Care Deliv. (Berlin, Heidelberg: Springer Press), 169–175. 10.1007/978-3-642-39982-4_16

